# Laughing During Conversations, But Not in Response to Digital Media, Is Associated With Less Loneliness, Social Isolation, and Social Withdrawal: A Cross‐Sectional Study of German Adults in 2023

**DOI:** 10.1002/hsr2.71354

**Published:** 2025-10-28

**Authors:** André Hajek, Angelina R. Sutin, Nicola Veronese, Pinar Soysal, Louis Jacob, Martina Luchetti, Razak M. Gyasi, Karel Kostev, Supa Pengpid, Karl Peltzer, Antonio Terracciano, Hans‐Helmut König

**Affiliations:** ^1^ Department of Health Economics and Health Services Research, Hamburg Center for Health Economics University Medical Center Hamburg‐Eppendorf Hamburg Germany; ^2^ College of Medicine Florida State University Tallahassee Florida USA; ^3^ Geriatrics Section, Department of Internal Medicine University of Palermo Palermo Italy; ^4^ Department of Geriatric Medicine, Faculty of Medicine Bezmialem Vakif University Istanbul Turkey; ^5^ Assistance Publique ‐ Hôpitaux de Paris (AP‐HP), Université Paris Cité, Lariboisière‐Fernand Widal Hospital Department of Physical Medicine and Rehabilitation Paris France; ^6^ Inserm, Université Paris Cité, U1153, CRESS Epidemiology of Ageing and Neurodegenerative Diseases (EpiAgeing) Paris France; ^7^ Research and Development Unit, Parc Sanitari Sant Joan de Déu CIBERSAM, ISCIII Sant Boi de Llobregat Barcelona Spain; ^8^ African Population and Health Research Center Nairobi Kenya; ^9^ National Centre for Naturopathic Medicine, Faculty of Health Southern Cross University Lismore New South Wales Australia; ^10^ University Hospital Marburg Philipps‐University Marburg Marburg Germany; ^11^ Department of Health Education and Behavioral Sciences, Faculty of Public Health Mahidol University Bangkok Thailand; ^12^ Department of Public Health Sefako Makgatho Health Sciences University Pretoria South Africa; ^13^ Department of Medical Research, China Medical University Hospital China Medical University Taichung Taiwan; ^14^ Department of Psychology University of the Free State Bloemfontein South Africa; ^15^ Department of Psychology, College of Medical and Health Science Asia University Taichung Taiwan

**Keywords:** hikikomori, laughing, loneliness, social exclusion, social isolation, social withdrawal

## Abstract

**Background and Aims:**

Due to the limited knowledge in this study area, we aimed to investigate the association of frequency of laughter with loneliness, social isolation (perceived and objective social isolation), and social withdrawal.

**Methods:**

Cross‐sectional data were from an online German sample of adults aged 18–74 years (*n* = 5000 individuals; data collection in late summer 2023). The De Jong Gierveld tool was used to quantify loneliness. The Lubben Social Network Scale was used to quantify objective social isolation, and the Bude and Lantermann tool was used to measure perceived social isolation. The German version of the 25‐item Hikikomori Questionnaire was used to measure social withdrawal. A frequently used tool was applied to quantify (1) the frequency of laughter and (2) the occasion on which one laughs (conversations with people, consuming digital media, cultural events, reading, other).

**Results:**

Frequent laughter was significantly associated with less loneliness, social isolation (both objective and perceived), and social withdrawal. Notably, laughing during conversations and cultural events was consistently linked to these outcomes, while laughing while consuming digital media showed no significant association. Laughing while reading and laughing while doing other things were partly associated with the outcomes.

**Conclusion:**

A higher frequency of laughter was associated with lower levels of loneliness, social isolation, and social withdrawal. Laughing while having conversations with people and attending cultural events were associated with more social integration, whereas laughing while consuming digital media was not significantly associated with any outcomes. These findings suggest that laughter could help reinforce social connections and help address the challenges of loneliness, but these associations need further testing with interventions and longitudinal research.

## Summary

- First study: examining the link of laughter with loneliness, social isolation, and social withdrawal.
- Frequent laughter is associated with less loneliness, social isolation, and social withdrawal; laughing during conversations and cultural events was linked to outcomes, whereas laughing while consuming digital media showed no significant association.
- Such knowledge is important because social disconnectedness is associated with unfavorable health outcomes such as morbidity and mortality.

## 1 Introduction

According to Gonot‐Schoupinsky et al. [1, pp. 4–5], “laughter is predominantly a physical behaviour, occurring alone or socially. It is often used as a form of verbal expression or communication.” It is often accompanied by a change in facial expression and laughing noises. It serves as a means of communication, often spontaneous or prompted, and aids personal development and social bonding [1]. It can evoke or be influenced by humour, playfulness, motives, emotions, circumstances, and cultural or individual differences [1].

Studies from Japan have shown that some people laugh out loud almost every day, whereas others almost never laugh. For example, one study using data from the Fukushima Health Management Survey showed that 27.1% of individuals aged 20 years and over laughed “almost every day,” whereas 12.9% laughed “almost never” [2]. Another study of adults 65 and older from the Japan Gerontological Evaluation Study showed that 36.7% of male and 47.2% of female laughed almost every day [3].

Numerous previous studies have shown that the frequency of laughing is important for various health outcomes, such as oral health [4], frailty [5], depression [5], functional disability [6], high blood pressure [7], and even mortality [8]. This positive effect of laughing is attributed to, among other things, an improvement in immune function and inflammation reduction [9, 10] resulting from positive emotions associated with laughter. Additionally, laughing at least once a week is associated with higher work engagement scores compared to laughing less than once a month [11]. One study showed a stress‐buffering effect of the frequency of laughter on the link between stressful events and stress symptoms [12]. Apart from physical and mental health, it is likely that frequency of laughter is also associated with metrics of social health, such as loneliness (a negative emotion that there is a difference between actual and desired social relationships, either qualitatively or quantitatively [13]), objective social isolation (lack of actual social activities, see Hajek and König [14]), perceived social isolation (feeling that one does not belong to the society [15]) and social withdrawal (referring to a behavioral tendency to isolate from key peer groups and to avoid social activities [16]).

Based on cross‐sectional data from the Japan Gerontological Evaluation Study, a previous study showed that living alone and inadequate social relationships (particularly quantified by meeting friends infrequently) were associated with a lower frequency of laughter [17]. Another cross‐sectional study based on data from the Fukushima Health Management Survey (individuals aged 20 years and older) found an association between a higher number of family members and a higher frequency of laughter [2]. Older Japanese individuals with hobbies also reported higher laughing frequency compared to those without hobbies [18]. Moreover, older individuals in Japan with a higher frequency of social participation tended to laugh more often [19]. Another study from Japan showed that greater social participation (women only) and social relationships (women and men) were associated with a higher frequency of laughter among older adults [3]. A few intervention studies have examined the effect of laughter therapy on loneliness, with mixed and inconclusive evidence. A small‐sample nonrandomized pilot study, for example, found that laughter therapy reduces loneliness among older adults living in nursing homes in Turkey [20]. A recent small sample RCT, however, showed that online laughter therapy sessions did not affect loneliness among nursing students during the COVID‐19 pandemic in Turkey [21]. A similar study likewise did not find an effect of online laughter therapy on the loneliness of healthy corporate employees in India during the pandemic. A positive effect on loneliness was found after a laughter yoga program among older adults living in a nursing home in Turkey [22]. Another pilot study investigated the impact of a new laughter therapy (1‐min laughie laughter prescription which refers to deliberate daily laughter) on the well‐being among 20 earthquake survivors in Turkey. Their descriptive analysis cautiously suggests positive effects of this prescription on loneliness [23].

The present research builds on this small but growing literature in several ways. First, we focus on the *general population in Germany* as opposed to older individuals (particularly from Japan) and the few small‐sample trials, mainly from Turkey. Second, due to *cultural differences* between Japan and Germany, there may be differences in the frequency and occasions of laughter. Third, we focus on *loneliness*, *social isolation*, and *social withdrawal* as opposed to social activities used in former observational research. Finally, in contrast to the few interventional studies that focus on specific therapeutic approaches (e.g., laughter yoga), we concentrate on laughter in everyday life, which is important in terms of ecological validity.

Due to the limited knowledge in this study area, our aim was to investigate the association between the frequency of laughter and loneliness, social isolation (both perceived and objective social isolation), and social withdrawal among the general adult population in Germany. We also examine how the occasion on which one laughs (e.g., with friends or when consuming digital media) is associated with these outcomes. Knowledge about such an association is important to characterize individuals susceptible to higher loneliness, isolation, or social withdrawal. Ultimately, such measures of social connection are associated with chronic conditions, suicidal ideation, perceived longevity, and mortality [24].

There are several reasons why there might be an association between the frequency of laughter and social metrics. For example, laughing while having conversations with other people (e.g., friends and relatives) may strengthen the emotional bond with others [25]. The release of endorphins associated with laughter may increase well‐being and reduce stress, which may contribute to feelings of social connection [26]. Furthermore, laughing can decrease fear and inhibitions, which may help individuals be more open and social.

On the other hand, if an individual laughs at a joke alone in front of the TV or a phone (and no one else laughs with them), such an individual may become more aware of being alone, miss other people, and feel isolated. Moreover, inappropriate laughter may sometimes be perceived as distasteful or even hurtful and may push other people back. For example, laughing at the expense of others may lead to marginalization if other people turn away from you as a result. Overall, however, we assume that the potential positive effects of laughter outweigh the negative effects. We also assume that laughter during conversations with other people will be positively associated with social factors, whereas laughing while consuming digital media may be less beneficial.

## 2 Materials and Methods

### 2.1 Sample

We used data from an online survey of a sample of *n* = 5000 individuals (18–74 years) residing in Germany, with data collection taking place from August to September 2023. Worth emphasizing that aged 18–74 years and living in Germany were the only two inclusion criteria. This implies that participants aged 17 and younger, as well as those aged 75 and older, were excluded from the study. Additionally, individuals living outside of Germany were also excluded. No other exclusion criteria were applied. The questionnaire was exclusively available in German. Consequently, it is very likely that individuals who were unable to read German chose not to participate.

The market research company Bilendi was responsible for collecting the data. Bilendi has its own online access panel. Various recruitment channels were employed for this online sample (including opinion platforms, search engine marketing, and partnership agreements). A quota‐based online sample was used to ensure a representative sample of the general adult population in Germany in terms of age, sex, and federal state. A random sample was selected from the online access panel population based on the aforementioned sociodemographic data.

Prior to participation in this study, all individuals provided their informed consent. Approval was given by the Local Psychological Ethics Committee at the University Medical Centre Hamburg‐Eppendorf (LPEK‐0629).

### 2.2 Outcomes of Social Disconnectedness

Hikikomori symptoms (denoting extreme social withdrawal) were assessed using the recently validated [27] German version of the 25‐item Hikikomori Questionnaire (HQ‐25) [28]. The final scores on the HQ‐25 range from 0 to 100, with higher scores signifying more social withdrawal (or increased hikikomori symptoms). Cronbach's *α* was 0.93 in this study. Loneliness was assessed using the De Jong Gierveld instrument (6‐item version) [29], which has scores between 0 and 6, with higher scores indicating higher levels of loneliness. Cronbach's *α* was 0.80 in the present study. Objective social isolation was quantified based on the Lubben Social Network Scale (LSNS‐6; 6‐item version [30]), which has scores between 0 and 30. The scale was reverse‐scored so that all outcomes could be interpreted in the same direction (i.e., the higher, the worse) with higher values indicating *higher* levels of objective social isolation. In this study, Cronbach's *α* was 0.87. Perceived social isolation was measured using an instrument created by Bude and Lantermann [15] (four items), with scores ranging from 1 to 4. Higher scores on the final scale reflect a higher level of perceived social isolation. Cronbach's *α* equaled 0.91 in this study.

### 2.3 Key Independent Variables

Following former research [31, 32], a single item was used to quantify the frequency of laughter referring to the frequency of laughing out loud (four categories: almost never; 1–3days/month; 1–5 days/week; almost every day).

Studies from Japan (e.g., Yamamoto et al. [33]) also quantified the occasions in which one laughs: talking with family or friends, watching TV or videos, looking at comics or magazines, listening to the radio, and watching comic stories and/or plays. We culturally adapted these occasions slightly, resulting in the following five occasions: conversations with people; consuming digital media (e.g., computer, smartphone, television); cultural events (e.g., cinema, theater); reading (e.g., newspapers, magazines); and other. Participants could select multiple occasions.

### 2.4 Covariates

Guided by previous research [34, 35], the sociodemographic covariates included sex (men; women; diverse), age (in years), marital status (single; divorced; widowed; living together: married or in partnership; living separately: married or in partnership), education based on CASMIN classification [36] (primary education; secondary education; tertiary education), labor force participation (full‐time employment; retired; other), and having a migration background (no or yes).

With regard to lifestyle‐related factors, the following covariates were chosen for regression analysis: smoking behavior (four categories which range from “never been a smoker” to “yes, daily”), alcohol consumption (six categories which range from “never” to “daily” alcohol consumption) and frequency of sports activities (five categories which range from “no sports activity” to “more than 4 h/week”).

Health‐related covariates were considered in regression analysis: Self‐rated health (single‐item, with 5 categories from 1 = *very poor* to 5 = *very good*), depressive symptoms, and chronic conditions. Depressive symptoms were assessed using the Patient Health Questionnaire‐9 (PHQ‐9) [37], ranging from 0 to 27 (higher scores reflect more depressive symptoms). The following 14 chronic conditions were also included as health‐related covariates: sleep disorder; thyroid disease; diabetes; asthma; heart disease (also heart failure, cardiac insufficiency); cancer; stroke; migraine; high blood pressure; dementia; joint disease (also arthrosis, rheumatism); chronic back problems; burnout; other illness.

### 2.5 Statistics

First, the sample is described overall and stratified by the frequency of laughter. Spearman's *ρ* was calculated to determine the association between the frequency of laughter and the outcomes. Unadjusted linear regression was used to examine the association of (1) frequency of laughter and (2) the occasion on which ones laugh with the outcomes. Adjusted linear regressions were used to examine these associations, first adjusted for sociodemographic factors, and then lifestyle‐ and health‐related factors. Robust standard errors were calculated. For the fully‐adjusted model, effect sizes (partial *η* ^2^ values) for the independent variables of interest were also reported. Partial *η* ^2^ values can be interpreted as follows [22]: 0.01 as “small,” 0.06 as “medium,” and 0.14 as “large.” Due to the forced response format, missing values were not present. The analyses were done with Stata 18.0 (Stata Corp., College Station, Texas), and the statistical significance was set at *p* < 0.05 (tests were two‐sided).

## 3 Results

### 3.1 Sample Characteristics

Characteristics of our sample overall and stratified by the frequency of laughter are in Table 1: 50.8% of participants were female and the average age was 46.9 years (SD: 15.3 years; range 18–74 years). Overall, 10.2% of the individuals reported laughing almost never, whereas 17.9%, 35.6%, and 36.3% reported 1–3 days/month, 1–5 days/week, and almost every day, respectively. Additionally, 83.0% of the individuals reported laughing when having “conversations with people,” while 64.8%, 31.8%, 34.2%, 13.5% answered during “consuming digital media,” during “cultural events,” during “reading,” and “other,” respectively. In the bivariate analysis, frequency of laughter was significantly associated with all other variables (except for sex and migration background).

**Table 1:** Sample characteristics (total sample and stratified by the frequency of laughter).

Variables	Laughing: almost never	Laughing: 1–3 days/month	Laughing: 1–5 days/week	Laughing: Almost every day	Total sample	*p*
*n* (%)	510 (10.2)	897 (17.9)	1780 (35.6)	1813 (36.3)	5000 (100.0)	
Laughing while having conversations with people: *N* (%)						
No	234 (45.9)	218 (24.3)	290 (16.3)	106 (5.8)	848 (17.0)	
Yes	276 (54.1)	679 (75.7)	1490 (83.7)	1707 (94.2)	4152 (83.0)	< 0.001
Laughing while consuming digital media: *N* (%)						
No	268 (52.5)	383 (42.7)	522 (29.3)	586 (32.3)	1759 (35.2)	
Yes	242 (47.5)	514 (57.3)	1258 (70.7)	1227 (67.7)	3241 (64.8)	< 0.001
Laughing while attending cultural events: *N* (%)						
No	433 (84.9)	670 (74.7)	1216 (68.3)	1091 (60.2)	3410 (68.2)	
Yes	77 (15.1)	227 (25.3)	564 (31.7)	722 (39.8)	1590 (31.8)	< 0.001
Laughing while reading: *N* (%)						
No	424 (83.1)	658 (73.4)	1200 (67.4)	1007 (55.5)	3289 (65.8)	
Yes	86 (16.9)	239 (26.6)	580 (32.6)	806 (44.5)	1711 (34.2)	< 0.001
Laughing while doing other things: *N* (%)						
No	410 (80.4)	802 (89.4)	1587 (89.2)	1524 (84.1)	4323 (86.5)	
Yes	100 (19.6)	95 (10.6)	193 (10.8)	289 (15.9)	677 (13.5)	< 0.001
Loneliness (DJG loneliness scale, 6‐item version; from 0 to 6, with higher scores reflecting higher loneliness levels): mean (SD)	4.4 (1.8)	3.9 (2.0)	3.2 (2.0)	2.3 (2.0)	3.1 (2.1)	< 0.001
Hikikomori symptoms (HQ‐25‐G; from 0 to 100, with higher scores reflecting more Hikikomori symptoms): mean (SD)	52.9 (16.7)	43.7 (15.8)	37.1 (16.9)	30.5 (16.8)	37.5 (18.1)	< 0.001
Objective social isolation (reversed LSNS‐6; 0–30, with higher scores reflecting lower social support/social engagement): mean (SD)	20.3 (6.0)	17.6 (5.6)	15.0 (5.5)	13.3 (5.8)	15.4 (6.1)	< 0.001
Perceived social isolation (Bude and Lantermann tool, from 1 to 4, with higher scores reflecting more perceived social isolation): mean (SD)	2.4 (0.9)	2.2 (0.8)	2.0 (0.8)	1.7 (0.8)	1.9 (0.8)	< 0.001
Sex: *N* (%)						
Male	283 (55.5)	421 (46.9)	881 (49.5)	866 (47.8)	2451 (49.0)	
Female	226 (44.3)	474 (52.8)	895 (50.3)	945 (52.1)	2540 (50.8)	
Diverse	1 (0.2)	2 (0.2)	4 (0.2)	2 (0.1)	9 (0.2)	0.06
Age: mean (SD)	51.1 (14.1)	47.7 (14.8)	45.3 (15.3)	46.8 (15.5)	46.9 (15.3)	< 0.001
Marital status: *N* (%)						
Single	173 (33.9)	276 (30.8)	490 (27.5)	394 (21.7)	1333 (26.7)	
Divorced	60 (11.8)	104 (11.6)	117 (6.6)	122 (6.7)	403 (8.1)	
Widowed	21 (4.1)	29 (3.2)	58 (3.3)	52 (2.9)	160 (3.2)	
Living together: married/partnership	246 (48.2)	458 (51.1)	1026 (57.6)	1163 (64.1)	2893 (57.9)	
Living separated: married/partnership	10 (2.0)	30 (3.3)	89 (5.0)	82 (4.5)	211 (4.2)	< 0.001
Education: *N* (%)						
Primary education	100 (19.6)	113 (12.6)	151 (8.5)	169 (9.3)	533 (10.7)	
Secondary education	319 (62.5)	562 (62.7)	1052 (59.1)	1054 (58.1)	2987 (59.7)	
Tertiary education	91 (17.8)	222 (24.7)	577 (32.4)	590 (32.5)	1480 (29.6)	< 0.001
Employment status: *N* (%)						
Full‐time employed	163 (32.0)	389 (43.4)	936 (52.6)	930 (51.3)	2418 (48.4)	
Retired	156 (30.6)	204 (22.7)	297 (16.7)	343 (18.9)	1000 (20.0)	
Other	191 (37.5)	304 (33.9)	547 (30.7)	540 (29.8)	1582 (31.6)	< 0.001
Migration background: *N* (%)						
No	462 (90.6)	794 (88.5)	1576 (88.5)	1617 (89.2)	4449 (89.0)	
Yes	48 (9.4)	103 (11.5)	204 (11.5)	196 (10.8)	551 (11.0)	0.58
Smoking status: *N* (%)						
Yes, daily	152 (29.8)	222 (24.7)	413 (23.2)	397 (21.9)	1184 (23.7)	
Yes, occasionally	25 (4.9)	84 (9.4)	208 (11.7)	199 (11.0)	516 (10.3)	
No, not anymore	155 (30.4)	242 (27.0)	455 (25.6)	514 (28.4)	1366 (27.3)	
Never been a smoker	178 (34.9)	349 (38.9)	704 (39.6)	703 (38.8)	1934 (38.7)	< 0.001
Alcohol consumption: *N* (%)						
Daily	34 (6.7)	41 (4.6)	92 (5.2)	108 (6.0)	275 (5.5)	
Several times a week	83 (16.3)	165 (18.4)	396 (22.2)	386 (21.3)	1030 (20.6)	
Once a week	54 (10.6)	158 (17.6)	355 (19.9)	331 (18.3)	898 (18.0)	
1–3 times a month	65 (12.7)	139 (15.5)	336 (18.9)	288 (15.9)	828 (16.6)	
Less often	103 (20.2)	232 (25.9)	356 (20.0)	402 (22.2)	1093 (21.9)	
Never	171 (33.5)	162 (18.1)	245 (13.8)	298 (16.4)	876 (17.5)	< 0.001
Frequency of sports activity: *N* (%)						
No sports activity	220 (43.1)	270 (30.1)	410 (23.0)	355 (19.6)	1255 (25.1)	
< 1 h/week	80 (15.7)	193 (21.5)	341 (19.2)	303 (16.7)	917 (18.3)	
1–2 h/week	87 (17.1)	208 (23.2)	467 (26.2)	440 (24.3)	1202 (24.0)	
2–4 h/week	73 (14.3)	130 (14.5)	320 (18.0)	366 (20.2)	889 (17.8)	
More than 4 h/week	50 (9.8)	96 (10.7)	242 (13.6)	349 (19.2)	737 (14.7)	< 0.001
Number of chronic conditions (from 0 to 14, with higher scores reflecting a higher number of chronic conditions): mean (SD)	2.1 (1.9)	1.7 (1.6)	1.4 (1.5)	1.4 (1.6)	1.5 (1.6)	< 0.001
Self‐rated health (from 1 = *very poor* to 5 = *very good*): mean (SD)	3.1 (0.9)	3.4 (0.8)	3.7 (0.8)	3.9 (0.8)	3.6 (0.8)	< 0.001
Depressive symptoms (PHQ‐9; from 0 to 27, with higher scores reflecting more depressive symptoms): mean (SD)	9.0 (6.9)	7.0 (5.5)	5.7 (5.2)	4.1 (4.6)	5.7 (5.4)	< 0.001

*Note: p* values are based on one‐way ANOVAs or *χ* ^2^ tests, as appropriate.

Abbreviation: SD = standard deviation.

Spearman's *ρ* for the association of frequency of laughing with the outcomes was: −0.34 with loneliness, −0.37, with social withdrawal, −0.34 with objective social isolation, and −0.29 with perceived social isolation (all *p*s < 0.001).

### 3.2 Regression Analysis

Results of unadjusted and adjusted linear regressions with frequency of laughter as the key independent variable are in Table 2. In both the unadjusted and adjusted models, even after adjusting for all confounders, compared to participants who reported laughing “almost never,” participants who reported laughing more frequently had more favorable outcomes: less loneliness, less social withdrawal, less objective social isolation, and less perceived social isolation. Partial *η* ^2^ values for frequency of laughter ranged from 0.02 (perceived social isolation) to 0.07 (social withdrawal). The associations were attenuated the most by the inclusion of health covariates but generally remained significant.

**Table 2:** Association of frequency of laughing with loneliness, social isolation and social withdrawal.

Independent variables	Loneliness				Objective social isolation				Perceived social isolation				Social withdrawal			
Frequency of laughter: (reference category: Almost never)																
1–3 days/month	−0.55*** (−0.75 to −0.35)	−0.55*** (−0.74 to −0.35)	−0.52*** (−0.72 to −0.32)	−0.16^+^ (−0.35 to 0.03)	−2.66*** (−3.29 to −2.02)	−2.17*** (−2.78 to −1.55)	−1.80*** (−2.41 to −1.20)	−1.43*** (−2.02 to −0.83)	−0.24*** (−0.33 to −0.15)	−0.27*** (−0.37 to −0.18)	−0.28*** (−0.37 to −0.18)	−0.08* (−0.16 to −0.00)	−9.18*** (−10.96 to −7.40)	−9.07*** (−10.83 to −7.31)	−8.27*** (−10.02 to −6.52)	−5.32*** (−6.97 to −3.66)
1–5 days/week	−1.25*** (−1.43 to −1.07)	−1.20*** (−1.38 to −1.02)	−1.14*** (−1.32 to −0.96)	−0.55*** (−0.73 to −0.37)	−5.25*** (−5.83 to −4.67)	−4.26*** (−4.83 to −3.70)	−3.68*** (−4.24 to −3.12)	−3.01*** (−3.56 to −2.45)	−0.45*** (−0.53 to −0.36)	−0.49*** (−0.57 to −0.40)	−0.49*** (−0.57 to −0.40)	−0.17*** (−0.24 to −0.09)	−15.76*** (−17.41 to −14.11)	−15.33*** (−16.98 to −13.69)	−14.11*** (−15.75 to −12.47)	−9.32*** (−10.90 to −7.74)
Almost every day	−2.12*** (−2.30 to −1.94)	−2.01*** (−2.19 to −1.83)	−1.94*** (−2.12 to −1.76)	−1.13*** (−1.31 to −0.94)	−6.98*** (−7.56 to −6.39)	−5.98*** (−6.55 to −5.41)	−5.37*** (−5.93 to −4.80)	−4.44*** (−5.02 to −3.87)	−0.74*** (−0.82 to −0.65)	−0.74*** (−0.83 to −0.66)	−0.74*** (−0.82 to −0.65)	−0.30*** (−0.37 to −0.22)	−22.33*** (−23.97 to −20.69)	−21.23*** (−22.88 to −19.59)	−19.95*** (−21.59 to −18.31)	−13.31*** (−14.93 to −11.70)
Sociodemographic covariates		✓	✓	✓		✓	✓	✓		✓	✓	✓		✓	✓	✓
Lifestyle‐related covariates			✓	✓			✓	✓			✓	✓			✓	✓
Health‐related covariates				✓				✓				✓				✓
Constant	4.45*** (4.29–4.60)	5.62*** (5.31–5.93)	5.80*** (5.46–6.13)	4.69*** (4.22–5.16)	20.30*** (19.78–20.82)	18.07*** (17.11–19.04)	20.53*** (19.49–21.56)	21.57*** (20.09–23.05)	2.40*** (2.32–2.48)	3.29*** (3.16–3.43)	3.29*** (3.14–3.44)	2.39*** (2.21–2.56)	52.85*** (51.40–54.30)	68.52*** (65.83–71.20)	73.89*** (71.04–76.74)	57.60*** (53.64–61.56)
Observations	5000	5000	5000	5000	5000	5000	5000	5000	5000	5000	5000	5000	5000	5000	5000	5000
*R* ^2^	0.12	0.17	0.19	0.31	0.13	0.21	0.25	0.27	0.08	0.20	0.22	0.46	0.15	0.22	0.25	0.37

*Note:* Findings of linear regressions. Unstandardized beta‐coefficients; 95% confidence intervals in parentheses. Sociodemographic covariates: sex, age, marital status, education, employment situation, and migration background; lifestyle‐related covariates: frequency of sports activities, smoking behavior, and alcohol consumption; health‐related covariates: self‐rated health, depressive symptoms, and count of chronic conditions.

*p < 0.05

**p < 0.01.

***p < 0.001.

^+^ *p* < 0.10.

Results of unadjusted and adjusted linear regressions with the occasion on which one laughs as the key independent variable are in Table 3. Laughing while having conversations with people and attending cultural events were consistently associated with all outcomes. Laughing while reading was significantly associated with loneliness and perceived social isolation in the fully adjusted model, and laughing while doing other things was significantly associated with perceived social isolation in the fully adjusted model. In contrast, laughing while consuming digital media was not significantly associated with any outcomes (it was only significantly associated with lower social withdrawal levels when adjusting for sociodemographic covariates/lifestyle‐related covariates). Partial *η* ^2^ values for laughing while having conversations with people ranged from 0.01 (perceived social isolation) to 0.07 (social withdrawal), followed by laughing while joining cultural activities (from 0.006 with loneliness to 0.02 with social withdrawal or objective social isolation). Partial *η* ^2^ values for occasions of laughter variables were consistently negligible for laughing while reading, consuming digital media, or doing other things (with all outcomes).

**Table 3:** Association of occasion of laughing with loneliness, social isolation, and social withdrawal.

Independent variables	Loneliness				Objective social isolation				Perceived social isolation				Social withdrawal			
Laughing while having conversations with people: Yes (ref.: No)	−1.27*** (−1.41 to −1.14)	−1.23*** (−1.36 to −1.09)	−1.16*** (−1.29 to −1.02)	−0.62*** (−0.75 to −0.49)	−3.17*** (−3.64 to −2.69)	−3.12*** (−3.56 to −2.67)	−2.94*** (−3.37 to −2.51)	−2.35*** (−2.79 to −1.91)	−0.54*** (−0.60 to −0.48)	−0.52*** (−0.58 to −0.46)	−0.49*** (−0.55 to −0.43)	−0.20*** (−0.25 to −0.15)	−17.25*** (−18.44 to −16.07)	−16.50*** (−17.67 to −15.33)	−15.76*** (−16.92 to −14.59)	−11.34*** (−12.46 to −10.22)
Laughing while consuming digital media: Yes (ref.: No)	0.08 (−0.04 to 0.20)	0.00 (−0.12 to 0.12)	−0.00 (−0.12 to 0.11)	0.04 (−0.06 to 0.15)	−0.07 (−0.41 to 0.28)	0.05 (−0.28 to 0.38)	0.07 (−0.25 to 0.40)	0.13 (−0.18 to 0.45)	0.04+ (−0.01 to 0.09)	−0.01 (−0.06 to 0.03)	−0.02 (−0.06 to 0.03)	0.01 (−0.03 to 0.05)	−0.37 (−1.35 − 0.60)	−1.24** (−2.18 to −0.30)	−1.19* (−2.12 to −0.25)	−0.75+ (−1.60 to 0.10)
Laughing while attending cultural events: Yes (ref.: No)	−0.46*** (−0.59 to −0.34)	−0.41*** (−0.53 to −0.29)	−0.37*** (−0.50 to −0.25)	−0.30*** (−0.41 to −0.19)	−2.62*** (−2.98 to −2.27)	−2.13*** (−2.47 to −1.79)	−1.82*** (−2.15 to −1.49)	−1.69*** (−2.02 to −1.37)	−0.12*** (−0.17 to −0.07)	−0.11*** (−0.16 to −0.07)	−0.11*** (−0.16 to −0.06)	−0.07*** (−0.11 to −0.03)	−6.50*** (−7.51 to −5.50)	−6.09*** (−7.06 to −5.13)	−5.54*** (−6.50 to −4.58)	−4.94*** (−5.81 to −4.07)
Laughing while reading: Yes (ref.: No)	−0.38*** (−0.51 to −0.26)	−0.29*** (−0.41 to −0.17)	−0.27*** (−0.39 to −0.15)	−0.17** (−0.28 to −0.06)	−0.01 (−0.36 to 0.33)	−0.36* (−0.70 to −0.03)	−0.28+ (−0.61 to 0.04)	−0.14 (−0.46 to 0.18)	−0.18*** (−0.23 to −0.13)	−0.10*** (−0.15 to −0.05)	−0.09*** (−0.14 to −0.05)	−0.04* (−0.08 to −0.00)	−2.31*** (−3.31 to −1.30)	−1.26* (−2.25 to −0.27)	−1.05* (−2.03 to −0.07)	−0.27 (−1.14 to 0.61)
Laughing while doing other things: Yes (ref.: No)	−0.12 (−0.28 to 0.04)	−0.16+ (−0.32 to 0.00)	−0.16* (−0.32 to −0.00)	−0.07 (−0.22 to 0.07)	0.61* (0.11 to 1.11)	0.07 (−0.41 to 0.56)	−0.12 (−0.60 to 0.36)	−0.02 (−0.49 to 0.45)	−0.13*** (−0.19 to −0.06)	−0.11*** (−0.17 to −0.05)	−0.10** (−0.16 to −0.04)	−0.05* (−0.10 to −0.00)	−1.38* (−2.69 to −0.06)	−1.49* (−2.75 to −0.24)	−1.73** (−2.97 to −0.49)	−0.95 (−2.11 to 0.21)
Sociodemographic covariates		✓	✓	✓		✓	✓	✓		✓	✓	✓		✓	✓	✓
Lifestyle‐related covariates			✓	✓			✓	✓			✓	✓			✓	✓
Health‐related covariates				✓				✓				✓				✓
Constant	4.44*** (4.29–0.59)	5.40*** (5.08–5.71)	5.63*** (5.28–5.98)	4.77*** (4.29–5.24)	18.85*** (18.34–19.37)	16.88*** (15.91–17.85)	19.73*** (18.68–20.79)	21.45*** (19.93–22.96)	2.47*** (2.40–2.54)	3.22*** (3.10–3.35)	3.23*** (3.09–3.38)	2.43*** (2.25–2.60)	55.11*** (53.81–56.41)	68.32*** (65.82–70.81)	74.34*** (71.52–77.17)	61.20*** (57.33–65.07)
*R* ^2^	0.08	0.13	0.15	0.30	0.09	0.19	0.23	0.26	0.08	0.19	0.20	0.45	0.18	0.24	0.27	0.39

*Note:* Findings of linear regressions. Unstandardized beta‐coefficients; 95% confidence intervals in parentheses. Sociodemographic covariates: sex, age, marital status, education, employment situation, and migration background; lifestyle‐related covariates: frequency of sports activities, smoking behavior, and alcohol consumption; health‐related covariates: self‐rated health, depressive symptoms, and count of chronic conditions.

* *p* < 0.05

** *p* < 0.01

*** *p* < 0.001.

^+^ *p* < 0.10.

## 4 Discussion

The aim of this study was to examine the association between frequency of laughter with loneliness, social isolation (both perceived and objective isolation), and social withdrawal. Key findings were that a higher frequency of laughter was associated with less loneliness, social isolation, and social withdrawal. Laughing while having conversations with people and attending cultural events were consistently associated with all outcomes. In contrast, laughing while consuming digital media was not significantly associated with any outcome. The effect size for the frequency of laughing varied from small to medium, depending on the outcome used. Laughing while having conversations with people had a medium effect size, followed by laughing while attending cultural events. The associations were most attenuated by the inclusion of health covariates. However, they generally remained significant. Using data from the *general adult population* in Germany, our present study extends current knowledge from studies focusing on *older adults* in Turkey and Japan.

Previous intervention studies partly demonstrated a positive effect of laughter therapy on loneliness among older adults residing in institutionalized settings [20]. Observational research from Japan also showed that a higher frequency and number of social participation was associated with a higher frequency of laughter among older adults [3, 19]. Our measures of social factors expand on this literature to include related yet distinct measures of social health (loneliness, social isolation, social withdrawal).

One key finding that laughing frequently was associated with the outcomes may be explained by the fact that laughing can improve emotional attachment, increase well‐being, reduce stress, and decrease fear and inhibition [26]. It is interesting and remarkable how different the occasions of laughter are associated with social health outcomes. Laughing during conversations and joining cultural events were associated with better social health. One function of laughter may be strengthening emotional bonds that support better social integration. Laughing in real life with other people (e.g., when attending cultural events or talking to other human beings) may be particularly beneficial. In contrast, laughing while consuming digital media (e.g., smartphone, television, or computer) was not significantly associated with any outcomes in our study. The positive effects of laughter (e.g., endorphin release) could be offset by negative effects (e.g., greater awareness of being alone, when assuming one is consuming digital media alone). However, further research is urgently needed. Our aforementioned potential explanation is particularly based on the assumption that individuals consume digital media alone—which is certainly not always the case. Of note, a recent study showed that the decrease in face‐to‐face communication is associated with reduced opportunities for laughter during the COVID‐19 pandemic (based on data from the Japan COVID‐19 and Society Internet Survey, individuals aged 15–79 years). However, this association was mitigated by telephone‐based and video‐based communication [38].

The association between laughing while consuming digital media and the outcomes used may also differ depending on whether it was consumed alone or with others. This could be another factor that may explain the missing association identified in our study. Furthermore, laughter alone may be viewed with scepticism and individuals may feel uncomfortable engaging with it, making them reluctant to embrace it (see also regarding solitary laughter [39]).

In our study, laughing while reading was significantly associated with less loneliness and perceived social isolation, but not with social withdrawal and objective social isolation in the fully‐adjusted model. One possible explanation could be that laughing when reading a book leads to closeness with the fictional or real characters from the books. Additionally, individuals may feel more connected to society if they read stories about society or its inhabitants and can laugh with them. Thus, if individuals feel more connected with the characters or with the society described in the book, or simply find reading enjoyable and entertaining, they are less likely to feel lonely or isolated.

Strengths and shortcomings of this study should be noted. Our study is the first to examine the relationship of frequency of laughter with loneliness, social isolation, and social withdrawal. Data were from a large sample of the general German adult population based on quotas for sex, age, and federal state. Established and validated instruments were used to measure the outcomes. Not only was laughter in general quantified, but also the situation of laughter. Nevertheless, it is difficult to clarify the directionality because cross‐sectional data were used. For example, loneliness is a distressing feeling and individuals who feel very lonely are probably unlikely to laugh. Thus, the distressing feeling of being lonely may contribute to a lack of laughter. Individuals may try to break out of a bad mood by doing an activity that makes them laugh. Thus, we recommend interventional and longitudinal studies. In addition, marginalized populations without access to the internet may not have participated in the survey. Moreover, the possibility of a self‐report bias cannot be dismissed. Additionally, it should be emphasized that the field of digital media covers a broad spectrum (and can also vary greatly in terms of quality).

## 5 Conclusion and Future Research

In conclusion, our study showed that a higher frequency of laughter was associated with less loneliness, social isolation (perceived and objective), and social withdrawal. Laughing, particularly during conversations or cultural events, was significantly associated with objective and subjective measures of social connection. In contrast, laughing while consuming digital media, such as when watching videos on TikTok or YouTube, did not help make people feel socially connected.

The findings of this study were based on a German sample, and we recommend that more research be conducted in other countries. It may be particularly important in low‐ and middle‐income countries, as it may have a favorable cost–benefit profile and be easy to implement. Furthermore, upcoming research could also clarify whether such associations between laughter and social disconnectedness differ in adolescents or the oldest old. Future qualitative studies could also be used to gain deeper insights into this link. Moreover, different laughing types (e.g., laughter of pleasure, laughter of social obligation, and laughter as relief from tension) could be explored in future research [40]. Similarly, future studies could measure the frequency of laughter by external assessment (e.g., trained observers) or other objective measures (audio signal processing techniques or by measuring facial muscle activity). This could help clarify whether the associations of interest differ by the assessment type, which was recently suggested [41]. More detailed research into the laughter situation (e.g., whether or not an individual is alone in laughing at digital media) could also be fruitful. In addition, future research could also focus on the relationship between laughter and solitude—which, unlike outcomes such as loneliness, could also be more positive. Future research could also focus on the link between laughter, humour and social factors based on existing theories such as the humour–laughter–affect model suggested by Gonot‐Schoupinsky [1]. Furthermore, the Solitary Laughter Model could be used as a theoretical foundation in future research in this field [42]. Upcoming research could investigate whether individuals laughing while consuming digital media tend to laugh more often in other situations. This, in turn, could be associated with social disconnectedness outcomes.

## Author Contributions

**André Hajek:** conceptualization, methodology, formal analysis, validation, writing – original draft, writing – review and editing, project administration, visualization, investigation. **Angelina R. Sutin:** conceptualization, visualization, writing – review and editing. **Nicola Veronese:** conceptualization, visualization, writing – review and editing. **Pinar Soysal:** conceptualization, visualization, writing – review and editing. **Louis Jacob:** conceptualization, visualization, writing – review and editing. **Martina Luchetti:** conceptualization, visualization, writing – review and editing. **Razak M. Gyasi:** conceptualization, visualization, writing – review and editing. **Karel Kostev:** conceptualization, visualization, writing – review and editing. **Supa Pengpid:** conceptualization, visualization, writing – review and editing. **Karl Peltzer:** conceptualization, visualization, writing – review and editing. **Antonio Terracciano:** conceptualization, visualization, writing – review and editing. **Hans‐Helmut König:** conceptualization, visualization, writing – review and editing, supervision, resources. All authors have read and approved the final version of the manuscript.

## Ethics Statement

Approval was given by the Local Psychological Ethics Committee at the University Medical Centre Hamburg‐Eppendorf (LPEK‐0629).

## Consent

Prior to participation in this study, all individuals provided their informed consent.

## Conflicts of Interest

The authors declare no conflicts of interest.

## Transparency Statement

The lead author André Hajek affirms that this manuscript is an honest, accurate, and transparent account of the study being reported; that no important aspects of the study have been omitted; and that any discrepancies from the study as planned (and, if relevant, registered) have been explained.

## Data Availability

The data that support the findings of this study are available on request from the corresponding author. The data are not publicly available due to privacy or ethical restrictions. André Hajek (lead and corresponding author) had full access to all of the data in this study and takes complete responsibility for the integrity of the data and the accuracy of the data analysis.
